# Blood Progenitor Cell Mobilization Driven by TWEAK Promotes Neovascularization and Reduces Brain Damage in a Rat Model of Intracerebral Hemorrhage

**DOI:** 10.3390/antiox14050601

**Published:** 2025-05-16

**Authors:** Daniel Romaus-Sanjurjo, Esteban López-Arias, Cristina Rodríguez, Pablo Hervella, Mariña Rodríguez-Arrizabalaga, Manuel Debasa-Mouce, Juan Manuel Pías-Peleteiro, Ramón Iglesias-Rey, Pablo Aguiar, Ángeles Almeida, José Castillo, Alberto Ouro, Tomás Sobrino

**Affiliations:** 1 NeuroAging Group Laboratory (NEURAL), Clinical Neurosciences Research Laboratory (LINC), Health Research Institute of Santiago de Compostela (IDIS), 15706 Santiago de Compostela, Spain; esteban.lopez.arias@sergas.es (E.L.-A.); marina.rodriguez.arrizabalaga@sergas.es (M.R.-A.); manuel.debasa@rai.usc.es (M.D.-M.); juan.manuel.pias.peleteiro@sergas.es (J.M.P.-P.); tomas.sobrino.moreiras@sergas.es (T.S.); 2 Centro de Investigación Biomédica en Red de Enfermedades Neurodegenerativas (CIBERNED), Instituto de Salud Carlos III, 28029 Madrid, Spain; pablo.aguiar.fernandez@sergas.es; 3 Translational Stroke Laboratory Group (TREAT), Clinical Neurosciences Research Laboratory (LINC), Health Research Institute of Santiago de Compostela (IDIS), 15706 Santiago de Compostela, Spain; 4 Institute of Biomedical Research of Salamanca (IBSAL), University Hospital of Salamanca, CSIC, University of Salamanca, 37007 Salamanca, Spain; c.rodriguez@usal.es (C.R.); aaparra@usal.es (Á.A.); 5 Institute of Functional Biology and Genomics (IBFG), CSIC, University of Salamanca, 37007 Salamanca, Spain; 6 Neuroimaging and Biotechnology Laboratory (NOBEL), Clinical Neurosciences Research Laboratory (LINC), Health Research Institute of Santiago de Compostela (IDIS), 15706 Santiago de Compostela, Spain; pablo.hervella.lorenzo@sergas.es (P.H.); ramon.iglesias.rey@sergas.es (R.I.-R.); jose.antonio.castillo.sanchez@sergas.es (J.C.); 7 Department of Neurology, Hospital Clínico Universitario, Universidad de Santiago de Compostela, 15706 Santiago de Compostela, Spain; 8 Molecular Imaging Biomarkers and Theragnosis Lab, Center for Research in Molecular Medicine and Chronic Diseases (CiMUS), University of Santiago de Compostela (USC), 15706 Santiago de Compostela, Spain; 9 Nuclear Medicine Service and Molecular Imaging Group, Health Research Institute of Santiago de Compostela (FIDIS), 15706 Santiago de Compostela, Spain

**Keywords:** endothelial progenitor cells, intracerebral hemorrhage, neovascularization, TWEAK

## Abstract

Non-traumatic intracerebral hemorrhage (ICH) is one of the most devastating and disabling forms of stroke; however, there are no effective pharmacological therapies available following the insult. Angiogenesis appears as a key step to overcoming the damage and promoting functional recovery. In this context, endothelial progenitor cells (EPCs) mobilization improves oxidative stress and promotes neovascularization, which has been linked to beneficial outcomes following both ischemic and hemorrhagic stroke. The TNF-like weak inducer of apoptosis (TWEAK), binding to its receptor Fn14, has been suggested as an inducer of EPCs differentiation, viability and migration to the injury site in a model of myocardial infarction. Here, we have performed a proof-of-concept preclinical study in a rat model of ICH where we report that a 50 μg/kg dose of rat recombinant TWEAK (rTWEAK) promotes blood progenitor cells mobilization, mainly EPCs. As soon as 72 h post-injury, brain neovascularization, and, importantly, long-term hematoma reduction and improved functional recovery is reported. In contrast, a higher dose of 150 μg/kg blocked those beneficial outcomes. Therefore, a low dose of rTWEAK treatment promotes neovascularization and reduces brain damage in a rat model of ICH. Further clinical studies will be needed to demonstrate if rTWEAK could represent a new strategy to promote recovery following ICH.

## 1. Introduction

Non-traumatic intracerebral hemorrhage (ICH) results from the spontaneous rupture of blood vessels in the brain and represents one of the most devastating and disabling forms of stroke. It accounts for 10–15% of all cases of stroke, showing the highest mortality rate: more than one-third of patients will not survive the first year [1]. Currently, the lack of effective therapies following ICH prevents better functional outcomes [2].

ICH induces cerebral angiogenesis around hematoma from 4 to 7 days post-lesion [3,4], a fact that has been related to motor recovery following ICH [5]. However, the exact role of endothelium during angiogenesis and neuronal repair following cerebral hemorrhage remains unknown, although endothelial progenitor cells (EPCs) have been suggested as the main players during these processes after stroke [6,7]. EPCs are circulating endothelial cells with the capacity to differentiate into mature endothelial cells and self-renewing [7]. EPCs are mobilized from their niches to the bloodstream following stroke, reaching the damaged area and carrying out neovascularization and endothelial repair through self-differentiation, paracrine signaling and exosomes [8,9,10]. Importantly, several clinical studies have reported an improved long-term recovery in stroke patients who had higher numbers of circulating EPCs within the first week after both ischemic [11,12,13] and hemorrhagic [14,15] insults. Furthermore, it has been reported that EPC mobilization reduces oxidative stress in several conditions including hypoxia [16,17]. Therefore, EPCs are a feasible therapeutic target following ICH.

The TNF-like weak inducer of apoptosis (TWEAK) is a ligand of the TNF family that can be presented in two forms: as a transmembrane protein and as a soluble ligand (sTWEAK) following furin proteases activity [18]. TWEAK was initially discovered in cell death-related mechanisms [19], but subsequent achievements revealed that it controls other activities besides apoptosis, such as proliferation, migration, differentiation, angiogenesis, and inflammation [20]. Indeed, the acute inflammatory response has an important role in ICH as shown by the association between several inflammatory biomarkers and a poor outcome following the injury [21]. The binding of sTWEAK to its cellular surface receptor Fn14 triggers several signaling pathways, including the canonical NFκB pathway [20]. The TWEAK-Fn14 axis regulates several physiological processes, and it is particularly important in tissue repair following acute damage [22].

Interestingly, Sheng and colleagues [23] highlighted the relevance of the TWEAK-Fn14-NFκB axis in EPC differentiation, viability, migration to injured tissue and angiogenesis in an in vivo model of acute myocardial infarction. Therefore, TWEAK-mediated mobilization of EPCs could represent a new avenue to promote recovery following ICH. However, the use of TWEAK may be controversial. Several previous studies suggested detrimental effects of endogenous sTWEAK as well as TWEAK treatments in both in vitro and in vivo models of cerebral ischemia [24,25,26,27]. There are no preclinical studies addressing the relationship between TWEAK treatment and hemorrhagic stroke so far.

Altogether, the main goal of this proof-of-concept preclinical study was to assess the use of TWEAK as a treatment to provide brain tissue repair through EPCs-mediated neovascularization in a rat model of ICH.

## 2. Materials and Methods

### 2.1. Animals

All Experimental protocols were approved by the University Clinical Hospital of Santiago de Compostela Animal Care Committee (15010/2019/004), according to the European Union (EU) rules (86/609/CEE, 2003/65/CE and 2010/63/EU) and the ARRIVE guidelines. Male adult Sprague Dawley (SD) rats (300–350 g) were kept in day/night cycles of 12/12 h at a mean temperature of 22  ±  1 °C and humidity of 60 ±  5%, and they had water and food ad libitum.

### 2.2. ICH Rat Model

The ICH rat model was used as previously described [28,29]. Prior to surgery, animals were injected subcutaneously with buprenorphine dosed at 0.05–0.1 mg/kg. Anesthesia was maintained by inhalation of 4% sevoflurane in a N_2_O/O_2_ mixture (70/30), and body temperature was maintained at 37 ± 0.5 °C with a heating pad until animals completely recovered from anesthesia and displayed normal motor activity. Rats were placed in a stereotaxic frame (Stoelting Co., Wood Dale, IL, USA) under sevoflurane anesthesia. After drilling a small burr hole, 1 μL of saline containing 0.2 U/μL bacterial collagenase type VII (Sigma-Aldrich Corp, St. Louis, MO, USA) was injected into the right striatum (0.6 mm anterior to bregma, −3.0 mm lateral and 5.5 mm depth) using a Hamilton syringe with a 30 G needle. Injections took 10 min, and the needle was left for an additional 10 min before removal. The burr hole was filled with bone wax (Ethicon, Somerville, NJ, USA), the scalp incision was closed with sutures, and lidocaine was applied to the wound locally.

### 2.3. Experimental Groups

Three experimental groups (n = 6 per group) were designated: (1) a control group treated with saline (0.9% of NaCl); (2) 50 group, treated with 50 μg/kg of rat recombinant TWEAK (rTWEAK, #80154-R01H, Sino Biological, Beijing, China) dissolved in saline; and (3) 150 group, treated with 150 μg/kg of rTWEAK dissolved in saline. The concentrations of rTWEAK were selected taking previous work as a reference [30], but here we wanted to assess potential dose-dependent effects. All treatments were given as a single bolus (jugular) at two-timepoints: (1) 1 h after ICH, following the basal magnetic resonance imaging (MRI), and (2) 24 h after ICH induction. The required sample size was calculated from previous studies using the same model in order to be able to detect a 25% effect size on hematoma growth versus controls (2-tailed *t*-test) [28,31]. Six animals per group are required to detect this difference with a power (1 − β) of 0.8 and α = 0.05. N was calculated using EPIDAT software version 4.2 (http://www.sergas.es/Saude-publica/EPIDAT-4-2, v4.2, accessed on 13 May 2025). Animals with hemorrhage located far from the basal ganglia (n = 2) were excluded from the study before treatment administration.

The experimental procedure was performed following several criteria derived from the STAIR (Stroke Therapy Academic Industry Roundtable) group guidelines for preclinical evaluation of stroke therapeutics [32]: (1) ICH hematoma was evaluated at 1 h, right before rTWEAK injections, by T2-weighted MRI to confirm ICH, as an index of the reliability of the hemorrhagic model; (2) animals were randomly assigned to treatment groups of the study; (3) researchers were blinded to treatment administration; (4) researchers were blinded to treatments during outcome assessment; and (5) temperature was controlled during the surgical period.

### 2.4. Magnetic Resonance Imaging Protocol

Based on a previous hematoma growth profile study [28,29], hematoma volumes were assessed basally (1 h after collagenase injection to induce ICH) and at 24 h, 7, 14, and 28 days after ICH induction by means of MRI conducted on a 9.4-T horizontal bore magnet system (Biospec 94/20USR, Bruker BioSpin, Ettlingen, Germany) with 20 cm-wide actively shielded gradient coils (440 mT/m), as previously described [28,29]. Before MRI acquisition, the animals were placed in a gas chamber containing 6% sevoflurane in a NO_2_/O_2_ mixture (70/30) until they were unconscious, and then they were positioned prone on the scanner bed. Rectal temperature was maintained at 37 ± 0.5 °C using a feedback-controlled heating pad. Radiofrequency transmission was achieved with a birdcage volume resonator, and the signal was detected using a four-element surface coil positioned over the head of the animal. Gradient-echo pilot scans were performed at the beginning of each imaging session for accurate positioning of the animal inside the magnet bore.

T2-weighted images were acquired using a Rapid Acquisition Relaxation Enhancement (RARE) sequence with the following acquisition parameters: echo time = 9.5 ms, 8 echos, rare factor = 4, repetition time = 3 s, number of averages = 2, field-of-view = 19.2 × 19.2 mm^2^, image matrix = 192 × 192 (isotropic in-plane resolution of 0.1 mm^2^/pixel), and 18 consecutive slices of 0.5 mm thickness. All images were processed using ImageJ (RasbandWS, ImageJ version 2.3, NIH, http://rsb.info.nih.gov/ij, 2.3v, accessed on 13 May 2025). The analyzed region of interest was the hematoma. Hematoma volumes (basal, 24 h and 7, 14, and 28 days), as well as edema volumes (24 h and 7, 14, and 28 days), were manually traced from T2-weighted images by a blind investigator.

Edema was firstly estimated by measuring the volumes of the affected (VLes) and contralateral (Vc) hemispheres and using the formula: edema (%) = 100 × [(VLes − Vc)/Vc]. Then, these values were normalized against those from the 24 h timepoint.

### 2.5. Bederson Scale

Following the STAIR criteria, animal models must show neurological and functional deficits in line with the produced lesion. The model of collagenase-induced hemorrhage primarily damages the striatum, producing a small forelimb paresis contralateral to the lesion. The neurological deficit was evaluated using a modified Bederson scale [33], ranging from 0 (asymptomatic) to 8 (severe deficit), which included the following items: spontaneous movement, spontaneous rotation, spontaneous flexing of the contralateral forelimb, edge detection, turn after tail suspension, and protection reflex.

Behavioral studies were performed at baseline (before surgery), as well as at 1, 7, and 28 days after ICH during the darkness cycle. An experienced blind investigator analyzed the behavioral tests.

### 2.6. Flow Cytometry Analysis of Blood Progenitor Cells

Blood samples were drawn from the tail vein before ICH (basal sample), and at days 1, 3, 7, 14, and 28 days after ICH. The samples were collected into K2EDTA tubes (BD Microtainer, BD, Franklin Lakes, NJ, USA), and then, erythrocytes were lysated using a commercial kit (FACS Lysing, #349202, BD Biosciences, USA). Immunofluorescence cell staining was performed with fluorescent conjugated antibodies anti-ckit (#567471, BD Biosciences, USA) and anti-sca-1 (#CL8934PE, Cederlane, Burlington, ON, Canada) [34]. Cell fluorescence was measured 15 min after staining by flow cytometry with BD FACS Aria II (BD, Franklin Lakes, NJ, USA). Numbers of EPCs (ckit^+^/sca1^+^) were calculated using the FACSDiva software (https://www.bdbiosciences.com/en-us/products/software/instrument-software/bd-facsdiva-software accessed on 13 May 2025), as previously described [34].

### 2.7. Tissue Processing

After the completion of the neuroimaging study, three animals per group at 28 days after ICH were euthanized by an overdose of anesthetic (sevoflurane 8%) and perfused with PBS and 4% formaldehyde. Brains were dissected out coronally in three parts and postfixed, in the same fixative solution, overnight at 4 °C. Brain blocks were rinsed with 0.1 M phosphate buffer and sequentially immersed in 10%, 20%, and 30% (*w*/*v*) sucrose in phosphate buffer until they sank. After cryoprotection, 20 μm-thick coronal sections were obtained with a freezing-sliding cryostat (Leica CM 1950 AgProtect; Leica Microsystems, Wetzlar, Germany).

### 2.8. Immunofluorescence Protocol

Sections were rinsed in 0.1 M phosphate buffer (PB) and incubated in 50 mM NH4Cl for 30 min. Then, a permeabilization protocol was carried out with 0.3% Triton X-100 (Sigma) in 0.1 M Tris/HCl (pH 8.0) for 10 min. Incubation in the primary antibody solution was carried out in CaCl_2_-containing buffer (0.1 mM CaCl_2_, 0.1 mM MgCl_2_, 0.1 mM MnCl) and blocking solution, 0.05% (*v*/*v*) Triton X-100 (Sigma) and 2% (*v*/*v*) goat serum (Jackson Immunoresearch Laboratories, West Grove, PA, USA) [35]. Rabbit anti-Iba1 (#019-19741, 1:200, Wako Chemicals, Neuss, Germany) and anti-IB4 (#L2140, 1:50, Sigma) primary antibodies were used. Sections were incubated for 2 h at room temperature with fluorophore-conjugated secondary antibodies (1:500, Jackson Immunoresearch Laboratories). Nuclei were stained with the commercial monomeric cyanine nucleic acid stain TO-PRO-3 (far-red fluorescence; Molecular Probes T3605, Invitrogen) for 10 min. After rinsing with PB, sections were mounted with Fluoromount (Sigma) aqueous mounting medium. Sections were examined with a spectral laser confocal microscope (Leica TSC-SL; Leica Microsystems) with three lasers: multiline Argon (488 nm), Helium-Neon (543 nm), and Helium-Neon (633 nm), and equipped with Å~ 40, Å~ 63 (1.4) HCX PL Apo oil immersion objectives for high-resolution imaging.

### 2.9. Immunofluorescence Quantifications

To quantify the intensity of each immunofluorescence (IF) signal in perilesional cortical regions, the area occupied by IB4+ vessels and Iba1+ was estimated using ImageJ software. All values were normalized against control values. The experimenter was blinded during quantifications.

### 2.10. Statistical Analyses

Data were presented as mean  ±  S.E.M. Normality of the data was determined by the Shapiro–Wilk normality test. The results of each experiment (lesion volume, EPCs numbers, immunofluorescence, and Bederson’s score) were analyzed by a one-way ANOVA (for normally distributed data) or a Kruskal–Wallis test (for non-normally distributed data). Correlation analysis was assessed with the Pearson correlation coefficient test. In the figures significant values were represented by different numbers of asterisks (vs. 150 μg/kg TWEAK treatment group) or pounds (vs Control group): *(#) *p*  <  0.05; **(##) *p*  <  0.01; ***(###) *p*  <  0.001; ****(####) *p*  <  0.0001. Statistical analysis was carried out using Prism 8 (GraphPad software, La Jolla, CA, USA).

## 3. Results

### 3.1. rTWEAK Decreases Long-Term Hematoma Volume After ICH Induction

**Figure 1:**
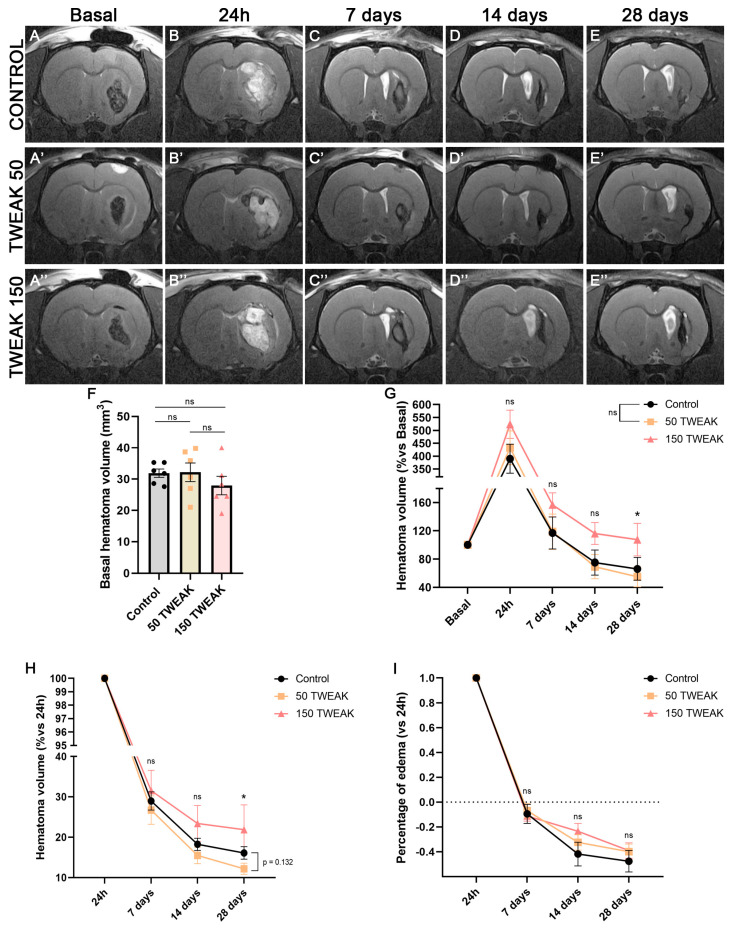
Treatment with 50 μg/kg of rTWEAK promotes a reduction in hematoma in the long term. (**A**–**E’’**) Analysis of hematoma volume was performed by T2-weighted magnetic resonance image. Saline (control) or rTWEAK (50 μg/kg and 150 μg/kg) treatments were administered at 1 and 24 h after collagenase injection. (**F**) Basal hematoma volumes. (**G**) Percentage of hematoma expansion from basal timepoint. (**H**) Percentage of hematoma reduction from the 24 h timepoint. (**I**) Analysis of edema evolution from the 24 h timepoint. In all graphics, results are expressed as the mean ± SEM and analyzed by one-way ANOVA or Kruskal–Wallis tests; differences from 150 group vs. 50 group are denoted as * *p*  <  0.05; non-significant results are denoted as “ns”. Six rats per group were used.

The intraparenchymal injection of collagenase caused an intracerebral hematoma with similar size in all animals at the basal timepoint (Figure 1A–A’’,F). The 50 µg/kg dose of rTWEAK showed decreased hematoma at long-term compared with both controls and the 150 μg/kg TWEAK treatment group, although differences were statistically significant at 28 days post-injury only vs. the 150 μg/kg TWEAK treatment group (Kruskal–Wallis test, *p* = 0.040) (Figure 1B–E’’,G). In order to investigate the effects of a subacute injection of the treatment (at 24 h), we also analyzed the reduction in brain damage in relation to this timepoint. Here, the long-term reduction in the 50 µg/kg dose is even clearer compared to both controls (Kruskal–Wallis test, *p* = 0.132) and the 150 µg/kg dose (Kruskal–Wallis test, *p* = 0.048) at 28 days post-ICH (Figure 1B–E’’,H). Edema was reduced in all experimental groups 7 days post-damage (Figure 1I); there was also a reduction in the volume of the ipsilateral hemisphere compared to the contralateral hemisphere (negative values) at 14 and 28 days, which was bigger in the controls than in the 50 and 150 μg/kg TWEAK treatment groups, but this was not statistically significant (Figure 1I).

### 3.2. rTWEAK Promotes and Maintains Long-Term Blood Progenitor Cell Mobilization

**Figure 2:**
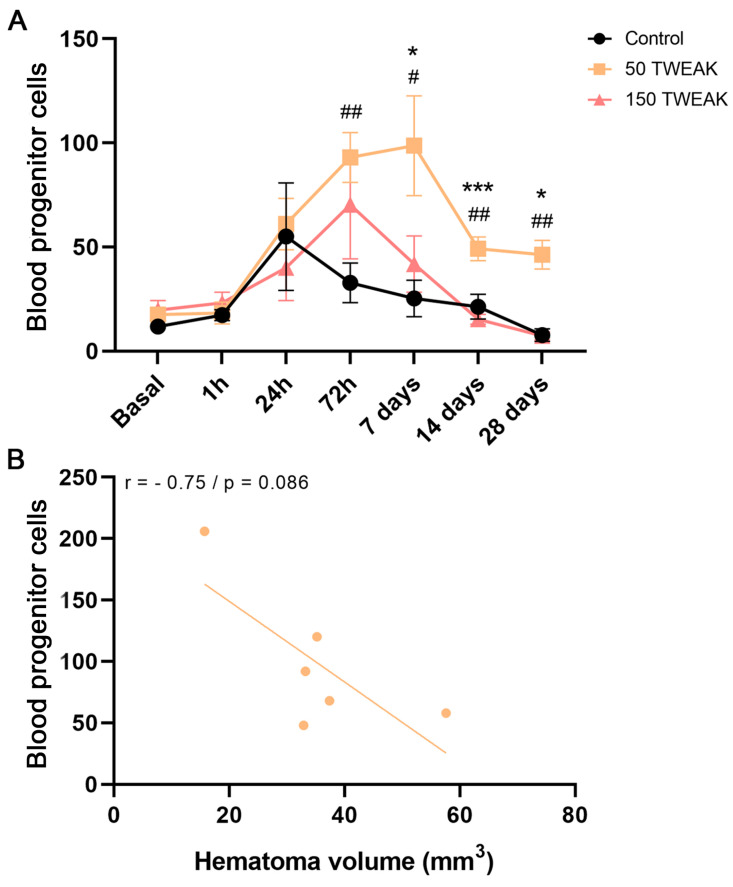
(**A**) Time course of blood progenitor cells numbers from blood samples (250.000 cells counted in each one). Circulating cells were determined by the expression of the surface antigens ckit^+^/sca1^+^ using flow cytometry: we first gated sca1^+^ peripheral blood cells and then examined the resulting population for dual expression of c-kit. Stats at each timepoint are controls (#) and 150 (*) vs. 50 group. Results are expressed as the mean ± SEM and analyzed by one-way ANOVA or Kruskal–Wallis tests (*(^#^) *p*  <  0.05; ^##^ *p*  <  0.01; *** *p*  <  0.001); non-significant results are denoted as “ns”. (**B**) Negative correlation between the number of blood progenitor cells and hematoma volume at 7 days post-injury (Pearson correlation’s coefficient, r = −0.75; *p* = 0.086). Six rats per group were used.

Our analysis demonstrates that only the 50 µg/kg treatment increased the levels of circulating blood progenitor cells, mainly EPCs, at different post-ICH timepoints (Figure 2). Such elevated numbers of EPCs were statistically significant as soon as 72 h post-injury (Kruskal–Wallis test, vs. Control: *p* = 0.009), and at 7 (One-way ANOVA test, vs. Control: *p* = 0.013; vs. 150: *p* = 0.045), 14 (One-way ANOVA test, vs. Control: *p* = 0.007; vs. 150: *p* = 0.0004), and 28 days (Kruskal–Wallis test, vs. Control: *p* = 0.004; vs. 150: *p* = 0.010) (Figure 2A). Moreover, the peak of circulating EPCs in the 50 μg/kg TWEAK treatment group was reached at 7 days post-injury (Figure 2A) and this correlates with lower hematoma volumes at this timepoint (Figure 2B).

### 3.3. rTWEAK Enhanced Cortical Neovascularization

**Figure 3:**
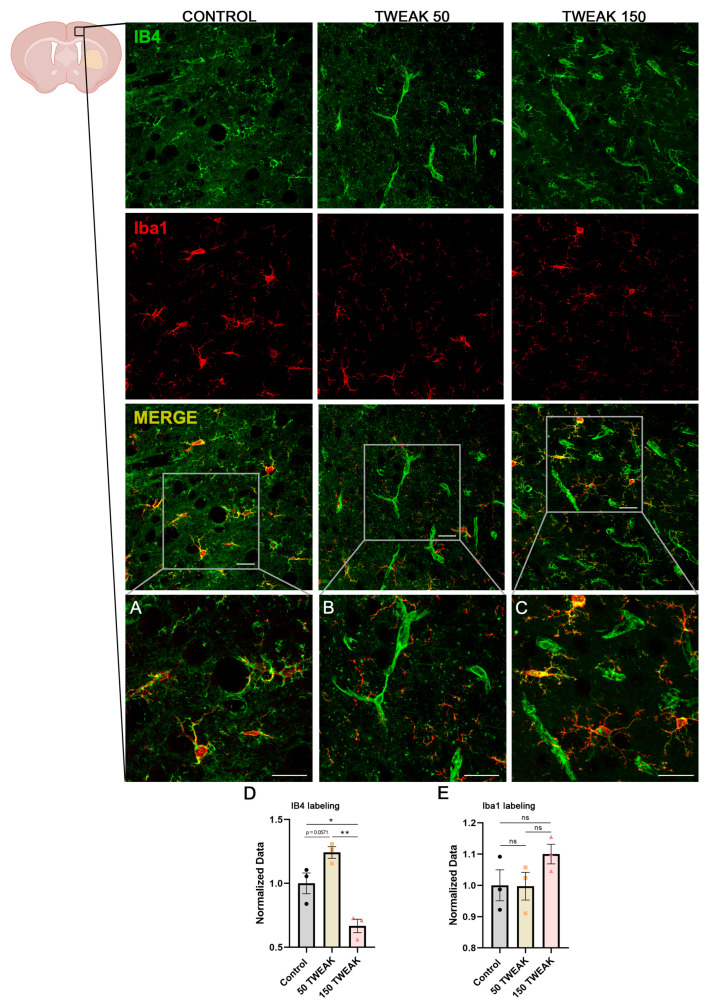
Microglia and vascular labeling. Saline (control, n = 3) or rTWEAK (50 μg/kg and 150 μg/kg, n = 3 each group) treated rats were subjected to experimental ICH. Brain sections were co-stained with the vascular endothelial cell marker IB4 and the microglia-specific marker Iba1 on day 28 after experimental ICH. Scale bars: 50 μm. (**A**–**C**) Detail from the cortical area (gray box) at higher magnification. Scale bars: 25 μm. Quantification of IB4 (**D**) and Iba1 (**E**) immunoreactivity in perilesional cortical regions. Results are expressed as the mean ± SEM and analyzed by one-way ANOVA test (* *p* < 0.05); (** *p* < 0.001); non-significant results are denoted as “ns”. Three rats per group were used.

**Figure 4:**
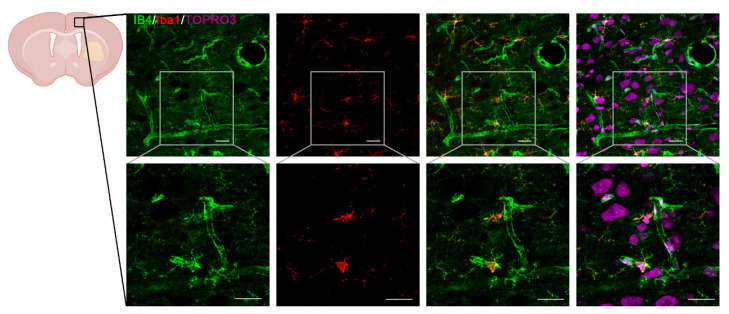
Neovascularization after ICH is increased only by the 50 µg/kg rTWEAK treatment. Brain sections from the 50 group were stained with the vascular endothelial cell marker IB4, the far-red fluorescent nuclear dye TO-PRO3 and the microglia-specific marker Iba1 on day 28 after experimental ICH. Scale bars: 50 μm. Gray box: Detail at higher magnification. Scale bars: 25 μm. Three rats per group were used.

We performed immunohistochemical analysis targeting the vascular cell marker isolectin-B4 (IB4), which represents a suitable index of vascularization [32,33]. Given that IB4 also labels microglial cells, we used the microglia-specific marker Iba1 to distinguish microglial cells from endothelial cells (Figure 3A–E). Regarding IB4+ cells, we observed that only the 50 μg/kg TWEAK treatment group had increased vascular density in cortical areas at 28 days post-injury, as revealed by the enhanced IB4 staining indicating vascular repair, and so, neovascularization (One-way ANOVA test, vs. Control: *p* = 0.0571; vs. 150: *p* = 0.001) (Figure 3B,E and Figure 4). Moreover, the 150 μg/kg TWEAK treatment group also displayed statistically significant differences compared to controls (One-way ANOVA test, *p* = 0.016). Iba1 staining displayed a 10% increase in the 150 μg/kg TWEAK treatment group compared to controls, but without statistical significance (Figure 3C,E).

### 3.4. The Effect of rTWEAK Treatments on Neurological Recovery

**Figure 5:**
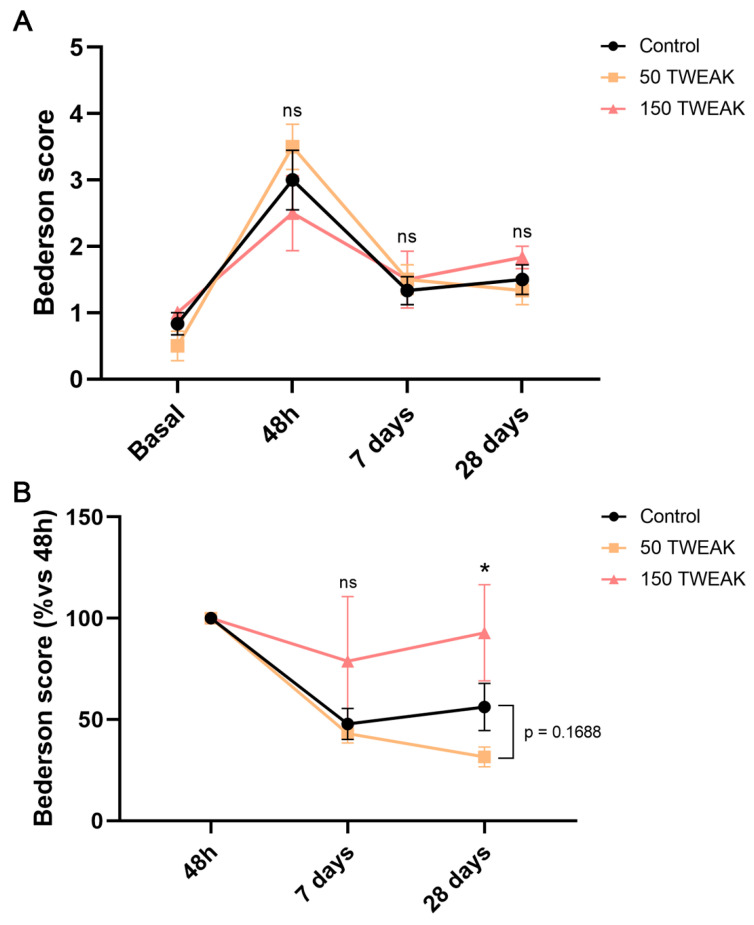
The 50 µg/kg rTWEAK treatments improved long-term neurological recovery after ICH. Neurological deficit was evaluated by the Bederson scale. (**A**) Bederson scores were obtained in the control and rats treated with the 50 µg/kg dose or the 150 µg/kg dose at 1 day before surgery (baseline) and 48 h, 7, and 28 days afterwards. (**B**) Analysis of neurological recovery from a 48 h timepoint. Results are expressed as the mean ± SEM and analyzed by the Kruskal–Wallis test (* *p* < 0.05); non-significant results are denoted as “ns”. Six rats per group were used.

We used the modified Bederson scale to assess any beneficial effect of rTWEAK treatments on the neurological deficits caused by the hemorrhagic lesion. Results showed that scores were close to 0 at baseline, as expected for healthy subjects (Figure 5A); however, both rTWEAK treatments showed higher deficit at 48 h (Figure 5A), in agreement with the larger hematoma volume seen at 24 h. Therefore, we analyzed the effects of a subacute injection (24 h) and observed that the 50 µg/kg dose, but not 150 µg/kg dose, induced a relevant neurological recovery at post-ICH timepoints compared to both other groups, especially at 28 days (Kruskal–Wallis test, vs. controls: *p* = 0.1688; vs. 150: *p* = 0.012) post-injury (Figure 5B).

## 4. Discussion

In this study, we show that a 50 µg/kg dose of rTWEAK induces smaller long-term lesion volumes, mobilizes higher numbers of circulating blood progenitor cells, mainly EPCs, and enhances neovascularization. Thus, our study represents the first proof-of-concept study assessing the therapeutic and dose-effect of TWEAK treatments in an animal model of ICH. Remarkably, our results suggest a direct effect of this treatment on EPCs-mediated vascularization. Here, we discuss the implications and possible mechanisms underlying this TWEAK-mediated response following ICH.

The role of the TWEAK-Fn14 axis as an in vitro and in vivo inducer of growth, proliferation, and migration of mature and progenitor endothelial cells is well-known, and it acts in a dose-dependent manner [23,25,36,37,38,39,40]. Intriguingly, our experiments showed that the rTWEAK treatment of 50 µg/kg mobilizes progenitor cells to the blood flow, likely EPCs, in a significant way, whereas the 150 µg/kg concentration had no effect on EPCs, likely because of the saturation of the Fn14 receptor. Moreover, several previous studies also showed that TWEAK can promote angiogenesis in mature and progenitor endothelial cells both in vitro and in vivo [23,36,37,39]. Our experimental group treated with a 50 µg/kg dose of TWEAK showed a higher degree of neovascularization in immunohistochemical analysis at day 28 post-injury, which agrees with previous studies. Based on the important role of EPCs in angiogenesis and neovascularization [7], it is plausible that a low dose of TWEAK induces EPCs-mediated neovascularization, given that this outcome was not seen in the high-dose group, where no significant EPCs mobilization was seen. Future studies should confirm whether TWEAK-mediated neovascularization is achieved through EPCs mobilization and/or angiogenic factors.

Addition to modulating angiogenesis and neovascularization, many studies have addressed the beneficial roles of EPCs following stroke such as reducing inflammation and promoting neuronal survival [7]. Indeed, higher numbers of circulating EPCs within the first week were associated with an improved long-term recovery in patients who suffered from both ischemic [11,12,13] and hemorrhagic [14,15] stroke. Here, we observed a higher and sustained EPCs mobilization and neovascularization after the administration of the 50 µg/kg dose, which was reflected in a beneficial long-term outcome both on lesion volume and on the neurological recovery of animals compared to control counterparts. Furthermore, peak levels of circulating EPCs in the 50 μg/kg TWEAK treatment group were reached at 7 days post-injury and were correlated with lower hematoma volumes. This result aligns with a previous clinical study of our group, in which hemorrhagic stroke patients with good functional outcome showed higher EPC levels at day 7, and a correlation was found between increased levels of EPCs and smaller ICH residual volume at 6 months [15]. Based on this, we proposed an indirect effect of TWEAK on reducing ICH damage through EPCs. As previously discussed, EPCs can promote angiogenesis and the repair of damaged vessels as well as trigger neovascularization. Moreover, a protective role of EPCs has been suggested by maintaining the integrity of the blood–brain barrier (BBB) following both ischemic [41] and hemorrhagic [15] stroke. Overall, our results highlight the positive role of increasing EPCs following stroke likely by restoring and protecting vascular integrity.

Results in rodent models of ischemic stroke showed that the blockage of the TWEAK/Fn14 axis results in beneficial effects [24,25,26,27]. Similarly, a few clinical studies reported a potential correlation between serum levels of sTWEAK and a poor functional outcome in ischemic stroke patients [42], as well as with the risk of developing early ICH growth [43]. Moreover, the ICH injury may not activate the same molecular pathways as the ischemic injury (e.g., the NFκB pathway [44]) [29,45], and this could explain the differences between previous ischemic studies and our results. Nevertheless, our strategy was not based on inhibiting TWEAK but on using it in a low dose given that several works indicated that the TWEAK/Fn14 axis can coordinate the inflammation and the response of progenitor cells in the context of acute tissue damage to promote tissue repair [46]. In this way, present results suggest that our hypothesis was partially right as the 50 µg/kg dose exhibited modest results compared to the control group regarding lesion, edema, and behavior, but it had a major impact on EPCs dynamics and angiogenesis. Remarkably, the second injection at 24 h post-injury appeared to be crucial in this TWEAK-mediated EPCs mobilization. Indeed, acute injection of rTWEAK at 1 h post-injury seems to slightly increase injury volumes at 24 h. Such results suggest that the subacute treatment is more efficient than the acute one, which may explain the statistically relevant differences when data are relativized to 24 h/48 h. Therefore, this indicates that activating the TWEAK/Fn14 axis following hemorrhagic stroke is not harmful per se, but it depends on the intensity and/or the timing. Further studies assessing lower doses than 50 µg/kg of TWEAK at different subacute/chronic timepoints would be necessary to find the best dose and time to apply the treatment.

TWEAK is a pro-inflammatory cytokine that controls other activities besides apoptosis, such as inflammation and oxidative stress [18,47,48,49,50]. A previous in vitro study reported the dose-dependent increase in pro-inflammatory cytokines released by astrocytes treated with TWEAK [51]. Similarly, we showed that only the 150 μg/kg TWEAK treatment group had an increase in microglia labeling on day 28 after ICH, suggesting increased neuroinflammation even in the chronic phase of the insult. Indeed, microglia plays vital roles in both tissue damage and repair processes after ICH, specifically the perihematomal activated microglia [52]. So, it is plausible that this increased inflammation seen in the 150 μg/kg TWEAK treatment group is linked to higher lesion volumes and sensorimotor deficit. Furthermore, excessive TWEAK-mediated activation increases reactive oxygen species in several cell cultures, including human umbilical vein endothelial cells [47], macrophages [48], and astrocytes [49]. Both neuroinflammation and oxidative damage are important causes of BBB disruption, as they alter the architecture of the neurovascular unit (NVU) and they exacerbate the brain injury following ICH as well [53]. Our data reported that a 150 µg/kg dose of TWEAK increased the hematoma volume compared to the control and the 50 µg/kg groups at all timepoints after ICH. Several in vivo studies showed glial cells as the main targets of endogenous TWEAK that trigger BBB dysfunction after cerebral ischemia and ICH [25,26,27,52,54,55]. Based on our data, we hypothesize that the 150 µg/kg dose is harmful enough to act on and activate NVU-forming glial cells, resulting in bigger lesion volumes and poorer animal behavior. Undoubtedly, further studies are mandatory to elucidate the exact impact of TWEAK on glial cells and BBB dysfunction following ICH.

There are several limitations in this study. We used the IB4 marker to assess angiogenesis as it is widely used as a well-known index of neovascularization [35,56]. Although additional assays assessing angiogenesis would have provided further information on this process, our proof-of-concept study fully supports the assumption that a 50 µg/kg dose of TWEAK promotes angiogenesis in vivo following ICH. This result agrees with previous studies showing that TWEAK activates the Hippo-YAP signaling [57], a positive regulator of angiogenesis [58], and promotes EPCs differentiation, viability, migration to injured tissue and angiogenesis in an in vivo model of acute myocardial infarction [23]. We cannot exclude the possibility of EPC-derived secretome/exosomes having a role during the neovascularization seen in this study [7,59], and future studies are needed to decipher the exact impact of these exosomes during this process. We characterized blood progenitor cells by studying the surface expression of both sca1 and ckit antibodies as previously described [34]. Although we did not determine the co-expression of those markers with CD31, a mature endothelial marker, ~90% of sca-1^+^ cells are also CD31^+^ [34], and so the majority of these cells are EPCs. Importantly, our results agree with those from hemorrhagic stroke patients, showing a correlation between increased levels of EPCs and smaller ICH residual volume at 6 months [15], similar to what we report here. Finally, the sample size may be considered a limitation as the 50 µg/kg dose exhibited modest results compared to the control group regarding lesion, edema, and behavior, although such differences are close to the statistical significance at day 28. However, this is a proof-of-concept preclinical study to assess the use of TWEAK as a treatment to provide brain tissue repair through EPCs-mediated neovascularization in a rat model of ICH, and present results suggest that our hypothesis is correct, as we observed a major impact on EPCs dynamics and angiogenesis.

In conclusion, we found that a 50 µg/kg dose of rTWEAK mobilizes higher numbers of circulating EPCs, enhances neovascularization, and induces smaller lesion volumes. Remarkably, our results suggest a direct effect of this treatment on EPC-mediated vascularization. However, further regulatory preclinical and clinical studies should be conducted to clarify whether rTWEAK may be able to be a therapeutic target in hemorrhagic stroke.

## Data Availability

Data are contained within the article.

## Abbreviations

The following abbreviations are used in this manuscript:

BBB	Blood–brain barrier
EPCs	Endothelial progenitor cells
ICH	Non-traumatic intracerebral hemorrhage
IF	Immunofluorescence
MRI	Magnetic resonance imaging
rTWEAK	Recombinant TWEAK
PB	Phosphate buffer
RARE	Rapid Acquisition Relaxation Enhancement
STAIR	Stroke Therapy Academic Industry Roundtable
TWEAK	TNF-like weak inducer of apoptosis
